# ΔNp63α induces the expression of FAT2 and Slug to promote tumor invasion

**DOI:** 10.18632/oncotarget.8696

**Published:** 2016-04-12

**Authors:** Tuyen T. Dang, Jill M. Westcott, Erin A. Maine, Mohammed Kanchwala, Chao Xing, Gray W. Pearson

**Affiliations:** ^1^ Harold C. Simmons Cancer Center, University of Texas, Southwestern Medical Center, Dallas, TX 75390-8807, USA; ^2^ McDermott Center for Human Growth and Disease, University of Texas, Southwestern Medical Center, Dallas, TX 75390-8807, USA; ^3^ Department of Pharmacology, University of Texas, Southwestern Medical Center, Dallas, TX 75390-8807, USA

**Keywords:** invasion, p63, FAT2, Slug, breast cancer

## Abstract

Tumor invasion can be induced by changes in gene expression that alter cell phenotype. The transcription factor ΔNp63α promotes basal-like breast cancer (BLBC) migration by inducing the expression of the mesenchymal genes Slug and Axl, which confers cells with a hybrid epithelial/mesenchymal state. However, the extent of the ΔNp63α regulated genes that support invasive behavior is not known. Here, using gene expression analysis, ChIP-seq, and functional testing, we find that ΔNp63α promotes BLBC motility by inducing the expression of the atypical cadherin FAT2, the vesicular binding protein SNCA, the carbonic anhydrase CA12, the lipid binding protein CPNE8 and the kinase NEK1, along with Slug and Axl. Notably, lung squamous cell carcinoma migration also required ΔNp63α dependent FAT2 and Slug expression, demonstrating that ΔNp63α promotes migration in multiple tumor types by inducing mesenchymal and non-mesenchymal genes. ΔNp63α activation of FAT2 and Slug influenced E-cadherin localization to cell-cell contacts, which can restrict spontaneous cell movement. Moreover, live-imaging of spheroids in organotypic culture demonstrated that ΔNp63α, FAT2 and Slug were essential for the extension of cellular protrusions that initiate collective invasion. Importantly, ΔNp63α is co-expressed with FAT2 and Slug in patient tumors and the elevated expression of ΔNp63α, FAT2 and Slug correlated with poor patient outcome. Together, these results reveal how ΔNp63α promotes cell migration by directly inducing the expression of a cohort of genes with distinct cellular functions and suggest that FAT2 is a new regulator of collective invasion that may influence patient outcome.

## INTRODUCTION

In mature organs, epithelial cells establish contacts with the adjacent extracellular matrix (ECM), become polarized, and form stable cell-cell adhesions that restrict cell migration [1–3]. The adoption of these differentiated traits is necessary for epithelial tissues to maintain the required three-dimensional architecture and carry out essential biological functions [4]. During tumor development, the aberrant activation of transcriptional regulatory networks can promote changes in cell state that induce epithelial derived neoplastic cells to invade into the stroma [5]. The induction of migratory behavior and local tumor invasion increases the potential for metastasis and correlates with reduced odds of patient survival [6]. Thus, we sought to define transcriptional control networks that confer tumor cells with invasive traits.

We focused on defining how the transcription factor ΔNp63α induces neoplastic cell motility. ΔNp63α is one of six isoforms encoded by the TP63 gene [7]. ΔNp63α is normally expressed in stem cells and basal cells [8] of epidermal [9] and epithelial tissue [10] where it is essential for cell proliferation, terminal differentiation and survival [9–13]. In human cancer, amplification of the TP63 gene and ΔNp63α expression are defining features of squamous tumors [14]. ΔNp63α is also expressed in invasive bladder cancer [15, 16] and basal-like breast cancer (BLBC) patients with poor outcome [17]. With respect to the control of invasive traits, ΔNp63α expression is increased during the initial induction of invasion in an orthotopic xenograft model [17] and is required for basal-like breast cancer (BLBC) cell migration [17]. ΔNp63α is also necessary for cellular protrusion formation in explants derived from a genetically engineered mouse (GEM) model of breast cancer [18]. Exogenous ΔNp63α can enhance the migration rate of esophageal squamous carcinoma cells [19] and ΔNp63α is required for head and neck squamous cancer cell migration as well [20]. ΔNp63α induced invasion is dependent on extrinsic factors. For example, BLBC cells that express ΔNp63α are reliant upon mammary fibroblasts to initiate ECM reorganization that permits collective invasion [17, 21]. Similarly, Luminal B type breast cancer cells expressing ΔNp63α are limited to invading into regions enriched in collagen I [18]. Thus, the invasive state induced by ΔNp63α is distinct from other types of invasive states, such as the mesenchymal-like trailblazer state that promotes invasion into a wide range of microenvironments, including those not permissive to ΔNp63α expressing tumor cell invasion [22]. Together, these results indicate that the ΔNp63α can promote a unique conversion in cell state that confers migratory ability.

ΔNp63α induces migration, in part, by promoting the expression of the transcription factor Slug and the tyrosine kinase Axl in BLBC cells [17]. Slug and Axl can initiate a transdifferentiation process called the epithelial-to-mesenchymal transition (EMT) that normally induces epithelial cells to transiently adopt a mesenchymal-like migratory state during embryonic development, tissue morphogenesis and wound healing [23–25]. During tumor development, the aberrant activation of EMT programs can lead to invasion and metastasis [26]. Interestingly, ΔNp63α expressing BLBC cells retain epithelial traits, such as E-cadherin expression, and do not undergo a complete transition to a mesenchymal state despite the induction of Slug and Axl [17]. This retention of epithelial character is potentially due to ΔNp63α simultaneously inducing the expression of miR205 [17], which can silence the E-cadherin suppressors ZEB1/2 [27, 28]. Thus, ΔNp63α promotes invasion, in part, by inducing a hybrid epithelial/mesenchymal state.

While the ΔNp63α dependent induction of Slug and Axl is critical for BLBC motility, exogenous Slug and Axl expression is not sufficient to promote the migration of ΔNp63α depleted BLBC cells [17]. This indicates that the induction of additional ΔNp63α targets is required to confer BLBC cells with a motile phenotype. To further understand how ΔNp63α induced migration, we used mRNA expression profiling of ΔNp63α depleted cells, chromatin immunoprecipitation coupled with next generation sequencing (ChIP-seq), and functional analysis of cell migration, to identify FAT2, SNCA, CA12, CPNE8 and NEK1 as ΔNp63α target genes that cooperate with Slug and Axl to promote motility. Further investigation of Slug and the atypical cadherin FAT2 as representative mesenchymal and non-mesenchymal genes revealed that they are co-expressed with ΔNp63α in patient tumors and required for ΔNp63α migration in multiple genetic contexts. Notably, mechanistic analysis determined that ΔNp63α and FAT2, influenced the establishment of cell-cell adhesions and were specifically required for the formation of cellular protrusions that precede collective invasion. Importantly, increased expression of ΔNp63α and FAT2 correlated with reduced odds of BLBC and NSCLC patient survival. Together, these results reveal how ΔNp63α promotes cell migration by inducing the expression of a cohort of mesenchymal and non-mesenchymal genes. Moreover these findings demonstrate that FAT2 is a new regulator of collective invasion that may be a biomarker for patient outcome.

## RESULTS

### Identifying genes that are positively regulated by ΔNp63α binding

**Figure 1:**
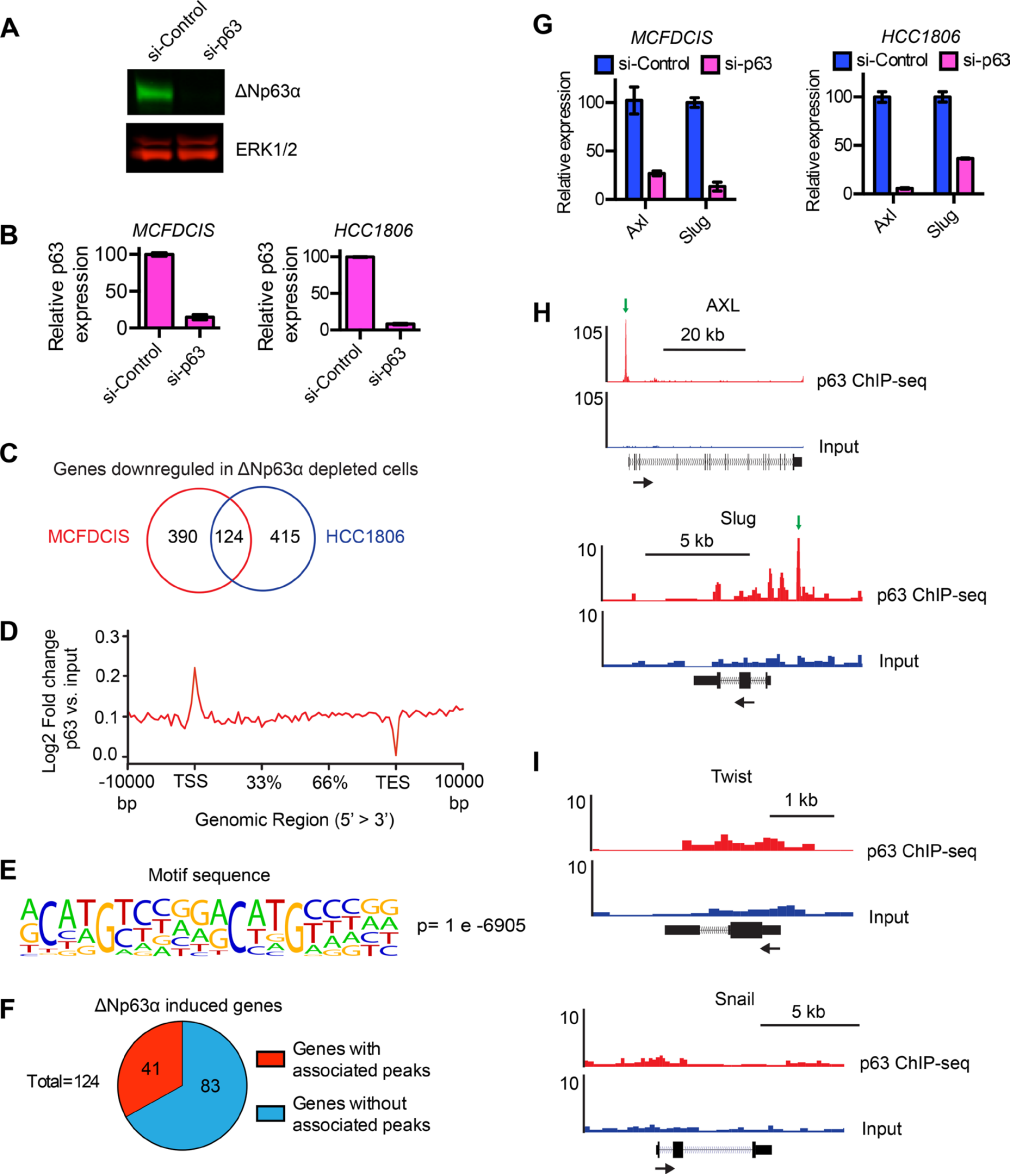
Identification of genes induced by ΔNp63α **A.** Representative immunoblot showing the expression of ΔNp63α and in MCFDCIS cells transfected with a control siRNA pool that does not target human genes or an siRNA pool that targets ΔNp63α. ERK1/2 expression is shown as a loading control. **B.** Relative expression of ΔNp63α mRNA in MCFDCIS and HCC1806 cells transfected with control or ΔNp63α siRNAs. **C.** Venn diagram shows the number of genes that were downregulated in MCFDCIS and HCC1806 cells in response to ΔNp63α depletion. Also see Supplementary Table S1 for the list of 124 genes that are decreased in expression in both p63-depleted populations. **D.** The ratio of the ΔNp63α ChIP-seq signal compared to the input DNA signal across all genes in the human genome. **E.** *De novo* analysis shows that the canonical ΔNp63α binding motif was the top-ranked motif enriched in ΔNp63α bound sequences. **F.** The number of ΔNp63α induced genes with ΔNp63α peaks within 2 kb of their TSS or associated enhancer regions. Also see Supplementary Tables S1 and S3 for the lists of genes induced by ΔNp63α. **G.** Relative expression of Axl and Slug mRNA in MCFDCIS and HCC1806 cells transfected with control (blue) or ΔNp63α (magenta) siRNAs. **H.** ΔNp63α ChIP-seq (red) and input DNA signals (black) in the genomic regions surrounding Axl and Slug. ΔNp63α binding sites are indicated with green arrows. The black arrows indicate gene orientation. Normalized read counts are indicated to the left of the tracks. **I.** Normalized ΔNp63α ChIP-seq (red) and input DNA signals (black) in the genomic regions surrounding Twist and Snail. No binding sites were detected. Normalized read counts are indicated to the left of the tracks.

We previously found that ΔNp63α promotes BLBC migration through the induction of a hybrid epithelial/mesenchymal state [17]. While these results provided insight into how ΔNp63α can regulate migration, they also indicated we had not yet defined the full complement of genes induced by ΔNp63α to confer a motile phenotype [17]. To better understand how ΔNp63α promotes migration, we began by analyzing the mRNA content of MCFDCIS and HCC1806 cells depleted of ΔNp63α by siRNAs (Figure 1A and 1B), as we had done previously to identify ΔNp63α-regulated genes [17]. We found that ΔNp63α depletion caused a 2-fold decrease (p < 0.05) in the expression of 124 genes in both the MCFDCIS and HCC1806 cells (Figure 1C and Supplementary Table S1). This indicated that ΔNp63α was necessary for the expression of a core set of 124 genes in migratory BLBC cells. ΔNp63α can also suppress gene expression, as indicated by the elevated expression of 128 genes in response to ΔNp63α depletion (Supplementary Table S2). However, we focused on determining how genes reliant on ΔNp63α for expression confer a motile phenotype based on our prior results showing that ΔNp63α promotes migration through the positive regulation of gene expression [17].

To prioritize ΔNp63α induced genes for further investigation, we performed ChIP-seq to determine which genes had ΔNp63α binding sites within 2 kb of their transcription start site (TSS) or associated enhancers. ΔNp63α binding within these regions has the potential to directly regulate gene expression based on previous investigations of ΔNp63α mechanism of action [29–33]. Indeed, analysis of the ΔNp63α ChIP-seq signal across all human genes showed an enrichment of ΔNp63α signal near TSSs (Figure 1D). The ΔNp63α ChIP-seq signal was also enriched in putative enhancer regions (Supplementary Figure S1A). In addition, the ΔNp63α bound sequences contained a canonical “CNNG” ΔNp63α binding motif (Figure 1E and Supplementary Figure S1B) that was defined in previous investigations of ΔNp63α binding specificity [34]. Thus, ΔNp63α binding in MCFDCIS cells was enriched in genomic regions that have the potential to direct gene expression and had the same sequence specificity found in other cell types. Notably, 41 of the 124 ΔNp63α-induced genes had ΔNp63α peaks within 2kb of their TSS or associated enhancer regions (Figure 1F, Supplementary Table S3), which suggested they were regulated directly by ΔNp63α. We therefore prioritized this set of 41 genes for further investigation.

The 41 ΔNp63α regulated genes included Slug and Axl (Figure 1G and Supplementary Table S3). This was consistent with our previous finding that ΔNp63α induced Slug and Axl expression to promote MCFDCIS and HCC1806 migration [17]. In addition, the ΔNp63α peak associated with Axl (Figure 1H) was within the same region of the Axl promoter that we had defined as a ΔNp63α binding site using ChIP-qPCR [17]. The ΔNp63α peak associated with Slug was a newly identified ΔNp63α binding site and was further confirmed by ChIP-qPCR (Figure 1H and Supplementary Figure S1C). Notably, this ΔNp63α binding site was located in the putative promoter region within 1 kb of the TSS, indicating that ΔNp63α may directly regulate Slug expression. Conversely, we did not detect ΔNp63α binding proximal to the EMT inducing transcription factors Snail or Twist (Figure 1I), consistent with our previous findings that ΔNp63α selectively regulates Axl and Slug expression to promote a hybrid state [17]. The remaining 39 genes were not previously implicated in ΔNp63α dependent cell migration. Together, our results revealed a cohort of genes that were positively regulated by ΔNp63α and contain associated ΔNp63α binding sites in putative regions of transcriptional regulation.

### ΔNp63α induces FAT2, CPNE8, SNCA, CA12 and NEK1 expression to promote breast cancer migration

**Figure 2:**
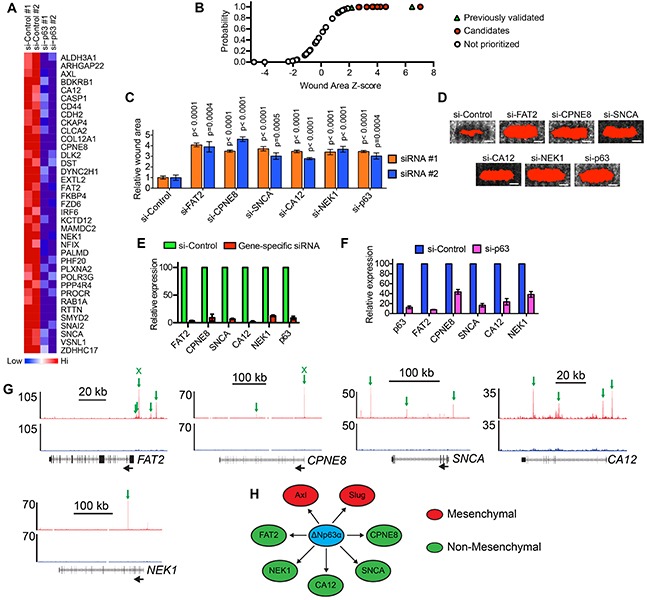
Determination of the ΔNp63α induced genes that are necessary for motility **A.** Heatmap shows the expression of 37 genes with associated ΔNp63α binding sites in MCFDCIS cells transfected with control or ΔNp63α siRNA pools. Biological replicates are shown. Also see Supplementary Figure S2 for a heatmap showing the expression of these 37 genes in HCC1806 cells. **B.** Cumulative distribution plot of the wound area z-scores for siRNAs targeting the 37 ΔNp63α activated genes shown in (A). Increasing z-score indicates increased wound area and potentially reduced migration. Green triangles indicate siRNAs targeting Axl and Slug, which were previously validated as ΔNp63α activated genes required for MCFDCIS wound closure. Red circles indicate siRNAs prioritized for further investigation. Clear circles indicate siRNAs that were not further tested. Also see Supplementary Figure S3A for a diagram outlining the wound closure assay testing methodology and Supplementary Table S5 for list of z-scores for each gene. **C.** Graph shows the wound area of MCFDCIS cells transfected with siRNAs targeting ΔNp63α and the indicated ΔNp63α regulated genes with z-scores >2 from (B). Two unique siRNA pools targeting the indicated genes were evaluated in distinct sets of experiments. Graph shows the mean ± standard deviation (SD). n= 6 wounds from 2 independent experiments for each siRNA pool. P-values were determined by unpaired Student's t-test. **D.** Representative images showing the wound closure of MCFDCIS cells transfected as indicated. Scale bar = 1 mm. The wound area, shown in red, is determined by fluorescent signal threshold analysis that defines cell-free space. Wound area images are from a representative experiment testing the siRNA #1 pools and were included in the quantification of wound closure. **E.** Confirmation that the siRNA #1 pools deplete target gene expression, as determined by qPCR. Green bars indicate relative expression in MCFDCIS cells transfected with a control siRNA pool that does not target human genes. Red bars indicate relative expression in MCFDCIS transfected with the siRNA #1 pool that targets the indicated gene. Graph shows mean ± range from 2 independent experiments. Also see Supplementary Figure S2B for the depletion of the indicated genes by the siRNA #2 pools. **F.** Expression of the indicated ΔNp63α activated genes in MCFDCIS cells transfected with a control or ΔNp63α siRNA pools, as determined by qPCR. Graph shows mean ± range from 2 independent experiments. **G.** ΔNp63α ChIP-seq (red) and input DNA signals (black) in the genomic regions surrounding the indicated ΔNp63α activated pro-migratory genes. ΔNp63α binding sites are indicated with green arrows. The black arrows indicate gene orientation. Normalized read counts are indicated to the left of the tracks. Also see Supplementary Figure S2C for the location of ΔNp63α bindings sites associated with the indicated genes in squamous carcinoma cells. Binding sites confirmed by ChIP-qPCR in Supplementary Figure S2D are indicated by an “x” above the arrow. **H.** Model depicting the direct regulation of mesenchymal (red) and non-mesenchymal (green) pro-migratory genes by ΔNp63α.

We next determined how genes that were potentially regulated by ΔNp63α binding influenced cell motility. To do this, we first identified siRNAs that targeted 37 of the 41 genes activated by ΔNp63α in MCFDCIS (Figure 2A) and HCC1806 cells (Supplementary Figure S2) and had associated ΔNp63α binding (Supplementary Table S4). We then determined the wound closure rates of MCFDCIS cells transfected with these 37 siRNAs in a one-condition/one-well format (Supplementary Figure S3). The siRNAs that targeted Axl and Slug, which we previously defined as ΔNp63α activated genes that promote migration [17], had z-scores >2 (Figure 2B and Supplementary Table S5). The 9 additional siRNAs with z-scores >2 were therefore prioritized for further investigation since they fell within the range of z-scores for 2 previously validated pro-migratory genes (Figure 2B and Supplementary Table S5). To reduce the chance of false-positives, the wound closure phenotype of MCFDCIS cells transfected with 2 unique siRNA pools targeting the 9 candidate genes was determined. Reproducible suppression of wound closure was detected in cells transfected with 2 distinct siRNA pools targeting FAT2, CPNE8, SNCA, CA12 and NEK1, (Figure 2C and 2D). Both unique siRNA pools depleted target gene expression by >60% (Figure 2E and Supplementary Figure S4A). The requirement of ΔNp63α for the expression of each gene was also confirmed by qPCR (Figure 2F). Analysis of peak locations revealed that there were multiple ΔNp63α binding sites associated with each pro-migratory gene, including binding sites outside of the initial prioritization parameters of being within 2 kb of TSSs or enhancer regions (Figure 2G). These additional peaks included ΔNp63α binding sites located 3-20 kb upstream of the FAT2 TSS (Figure 2G). ΔNp63α peaks were also detected within each of the 5 pro-migratory genes (Figure 2G). Analysis of a published dataset [29] revealed that ΔNp63α binding was detected in similar genomic locations relative to these genes in squamous carcinoma cells (Supplementary Figure S4B), indicating that ΔNp63α had the potential to regulate these pro-migratory genes in multiple genetic contexts. ΔNp63α binding associated with FAT2 and CPNE8 was additionally confirmed by ChIP-qPCR (Supplementary Figure S4C).

NEK1 is a serine/threonine kinase that coordinates cell cycle checkpoint control [35–37]; CPNE8 is a member of the copine family of lipid binding proteins [38]; SNCA (α-synuclein) is involved in synaptic transport [39]; FAT2 is an atypical cadherin [40] and CA12 is an exofacial carbonic anhydrase that can influence extracellular pH [41]. In contrast to Slug and Axl, these 5 genes are not canonical components of EMT programs. Thus, our findings provided new insight into the types of genes that are induced by ΔNp63α. Moreover, these results indicate that ΔNp63α can promote migration by increasing the expression of genes that function within distinct mesenchymal and non-mesenchymal signaling pathways with unique known functions (Figure 2H).

### ΔNp63α is co-expressed with FAT2 and Slug in patient tumors

**Figure 3:**
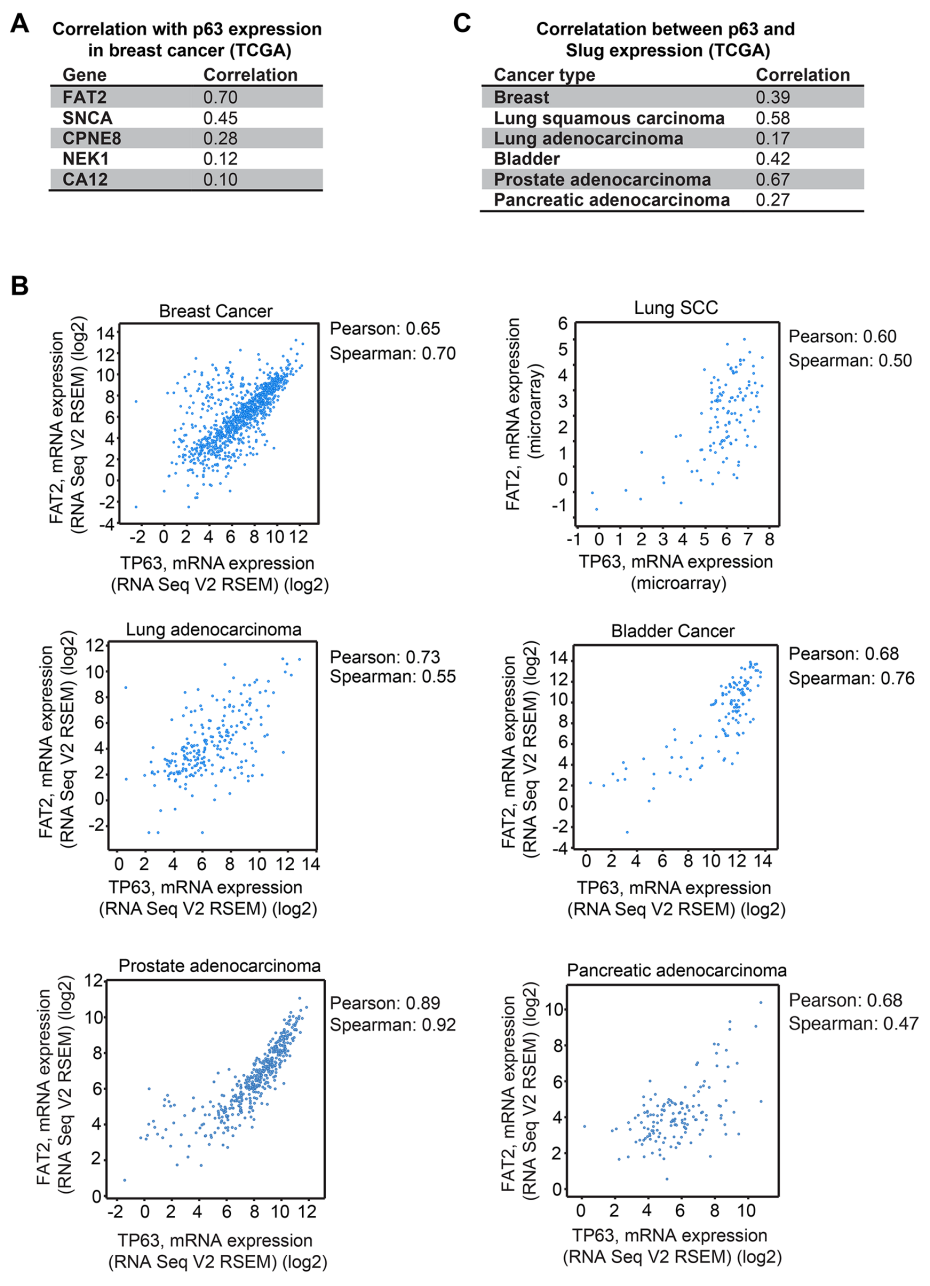
Correlation of ΔNp63α with FAT2 and Slug expression in patient tumors **A.** The Spearman correlation values for the co-expression of ΔNp63α with the indicated pro-migratory genes in breast cancer patient tumors are shown. Analysis was performed with cBioPortal on the provisional TCGA breast cancer RNA-seq dataset. **B.** The expression of ΔNp63α (x) and FAT2 (y) in breast cancer, lung squamous cell carcinoma, lung adenocarcinoma and bladder cancer. Analysis was performed with cBioPortal on the provisional TCGA datasets. **C.** The Pearson correlation values for the co-expression of ΔNp63α and Slug in the indicated tumors are shown. Analysis was performed with cBioPortal on provisional TCGA datasets.

To prioritize non-mesenchymal genes for further investigation, we determined how the expression of FAT2, SNCA, CPNE8, NEK1 and CA12 correlated with ΔNp63α mRNA levels in breast cancer patient tumors analyzed by TCGA [42]. FAT2 showed the strongest correlation with ΔNp63α (Figure 3A), so we further investigated the relationship between FAT2 and ΔNp63α expression in lung squamous cell carcinoma [43], lung adenocarcinoma [44], bladder cancer [45], prostate cancer [46] and pancreatic adenocarcinoma tumors analyzed by TCGA. Indeed, there was a strong correlation between ΔNp63α and FAT2 expression across the different tumor types (Figure 3B). In addition, we evaluated the relationship between ΔNp63α and Slug mRNA levels to determine if a similar correlation in expression could be detected between ΔNp63α and a canonical mesenchymal gene across tumor lineages. Consistent with our previous finding that ΔNp63α and Slug levels correlated in breast tumors using microarray expression data [17], a correlation between ΔNp63α and Slug expression was detected in breast tumors using RNA-seq data (Figure 3C). Importantly, ΔNp63α and Slug expression also strongly correlated in lung SCC, bladder cancer and prostate adenocarcinoma (Figure 3C). Thus, our results suggest that mesenchymal and non-mesenchymal genes that are induced by ΔNp63α to promote migration had the potential to contribute to the phenotypes of multiple types of ΔNp63α expressing primary tumors.

### ΔNp63α induced expression of FAT2 and Slug is necessary for lung SCC cell migration

**Figure 4:**
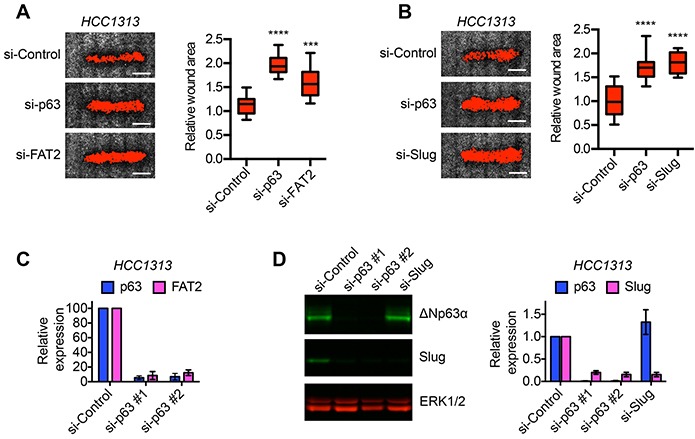
ΔNp63α dependent expression of FAT2 and Slug is required for lung SCC migration **A.** Representative images showing the wound closure of HCC1313 lung SCC cells transfected with control, ΔNp63α or FAT2 siRNAs. Scale bars = 1 mm. Box and whisker plots show the quantification of wound closure (n=12 wounds from 2 independent experiments). Whiskers indicate minimum and maximum. ***p< 0.001, ****p< 0.0001, unpaired Student's t-test. **B.** Representative images showing the wound closure of HCC1313 lung SCC cells transfected with control, ΔNp63α or Slug siRNAs. Scale bars = 1 mm. Box and whisker plots show the quantification of wound closure (n=12 wounds from 2 independent experiments). Whiskers indicate minimum and maximum. ****p< 0.0001, unpaired Student's t-test. **C.** Expression of ΔNp63α (blue) and FAT2 (magenta) in HCC1313 cells transfected with 2 distinct ΔNp63α siRNA pools as determined by qPCR. Graph shows mean ± range (n=2). **D.** Representative immunoblot showing the expression of ΔNp63α and Slug in HCC1313 cells transfected as indicated. Two distinct ΔNp63α siRNA pools (#1 and #2) were evaluated. ERK1/2 expression is shown as a loading control. Graph shows the quantification of ΔNp63α (blue) and Slug (magenta) expression as determined by immunoblotting (mean ± range, n=2).

To determine if the ΔNp63α dependent induction of FAT2 and Slug was a indeed a mechanism for the control of migration that extended beyond BLBC, we defined the requirement of ΔNp63α, FAT2 and Slug for migration in lung SCC cells. We chose lung SCC because ΔNp63α expression is a defining trait of lung SCC cells [14] and our results indicated that ΔNp63α was co-expressed with FAT2 and Slug in patient lung SCC. However, it was not known if ΔNp63α was required for lung SCC migration. Similar to our findings in BLBC cells, ΔNp63α depletion reduced the rate of HCC1313 lung SCC wound closure (Figure 4A), indicating that ΔNp63α can promote lung SCC migration. FAT2 and Slug were also required for HCC1313 migration (Figure 4A and 4B). Moreover, consistent with the strong correlation in expression detected in patient tumors, ΔNp63α was necessary for FAT2 and Slug expression in lung SCC cells (Figure 4C and 4D). Thus, our results indicate that FAT2 and Slug are part of a conserved ΔNp63α dependent gene expression program that is essential for migration in distinct tumor types.

### ΔNp63α dependent expression of FAT2 and Slug reduces the localization of E-cadherin to cell-cell adhesions

**Figure 5:**
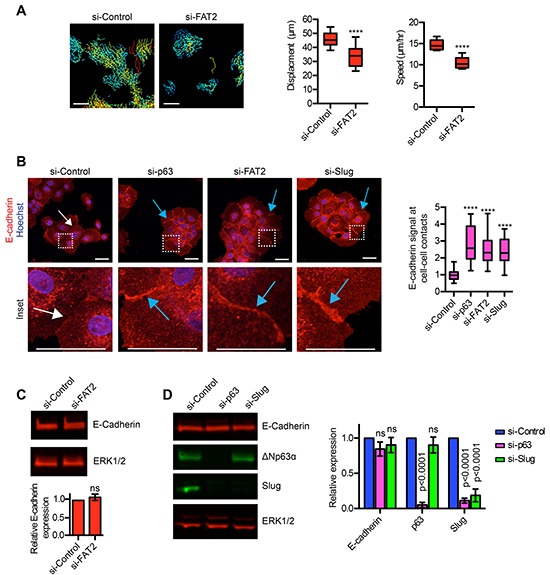
ΔNp63α dependent expression of FAT2 and Slug influences the formation of cell-cell contacts **A.** Time-lapse imaging performed on sub-confluent MCFDCIS cells transfected as with control or FAT2 siRNAs. Cells were imaged for 7 h to evaluate spontaneous cell motility. Tracking of spontaneous movement is shown. Each track indicates the movement of an individual cell with blue indicating slower velocity and red indicate faster velocity. Scale bars, 100 μm. Box and whisker plots show the quantification of cell displacement and speed (n=12 fields of view from 2 independent experiments). Whiskers indicate minimum and maximum. ****p< 0.0001, unpaired Student's t-test. **B.** MCFDCIS cells transfected as indicated were immunostained with α-E-cadherin antibody (red) and counterstained with Hoechst (blue, nuclei). Representative images are shown (n=4). White arrows indicate representative cell-cell contacts with low amounts of E-cadherin. Blue arrows indicate representative cell-cell contacts with increased amounts of E-cadherin compared to control cells. The inset regions are defined by the dashed white squares. Scale bars = 50 μm. Box and whisker plot shows the quantification of E-cadherin signal specifically in the region of cell-cell contacts (n= 30 or more cell-cell contacts from 4 independent experiments). Whiskers indicate minimum and maximum. ****p< 0.0001, unpaired Student's t-test. **C.** Representative immunoblot showing the expression of E-cadherin in MCFDCIS cells transfected with control or FAT2 siRNA pools. ERK1/2 expression is shown as a loading control. Graph shows the quantification of E-cadherin expression as determined by immunoblotting (mean ± SD, n=3). **D.** Representative immunoblot showing the expression of E-cadherin, ΔNp63α and Slug in MCFDCIS cells transfected with control or ΔNp63α or Slug siRNA pools. ERK1/2 expression is shown as a loading control. Graph shows the quantification of E-cadherin, ΔNp63α and Slug expression after normalization to total ERK1/2 levels as determined by immunoblotting (mean ± SD, n=5).

To better understand how ΔNp63α promotes motility, we further investigated how FAT2 and Slug influenced the migratory traits of MCFDCIS cells. We previously determined that ΔNp63α and Slug are necessary for spontaneous MCFDCIS motility [17]. Therefore, we determined if FAT2 also influenced spontaneous movement by performing time-lapse imaging on MCFDCIS cells transfected with FAT2 siRNAs. Indeed, FAT2 depletion reduced MCFDCIS cell displacement and speed (Figure 5A), similar to our prior analysis of ΔNp63α and Slug depleted cells [17]. The formation of stable cell-cell adhesions can restrict the spontaneous movement of sub-confluent cells [47], so we next determined how ΔNp63α, FAT2 and Slug influenced the formation of E-cadherin dependent intercellular adhesions. The cell-cell contacts formed by ΔNp63α, FAT2 and Slug depleted cells contained greater amounts of E-cadherin compared to the control MCFDCIS cells (Figure 5B), which suggests the presence of more completely assembled adhesion junctions. E-cadherin can be transcriptionally repressed to reduce cellular adhesion [48]. However, ΔNp63α, FAT2 and Slug depletion did not reduce total E-cadherin abundance (Figure 5C and 5D), indicating that ΔNp63α signaling regulates the formation of E-cadherin dependent cell-cell contacts through an alternative mechanism. Together, these results indicate that the ΔNp63α dependent induction of FAT2 and Slug prevents the establishment of mature cell-cell adhesions.

### ΔNp63α dependent induction of FAT2 and Slug is required for collective invasion

**Figure 6:**
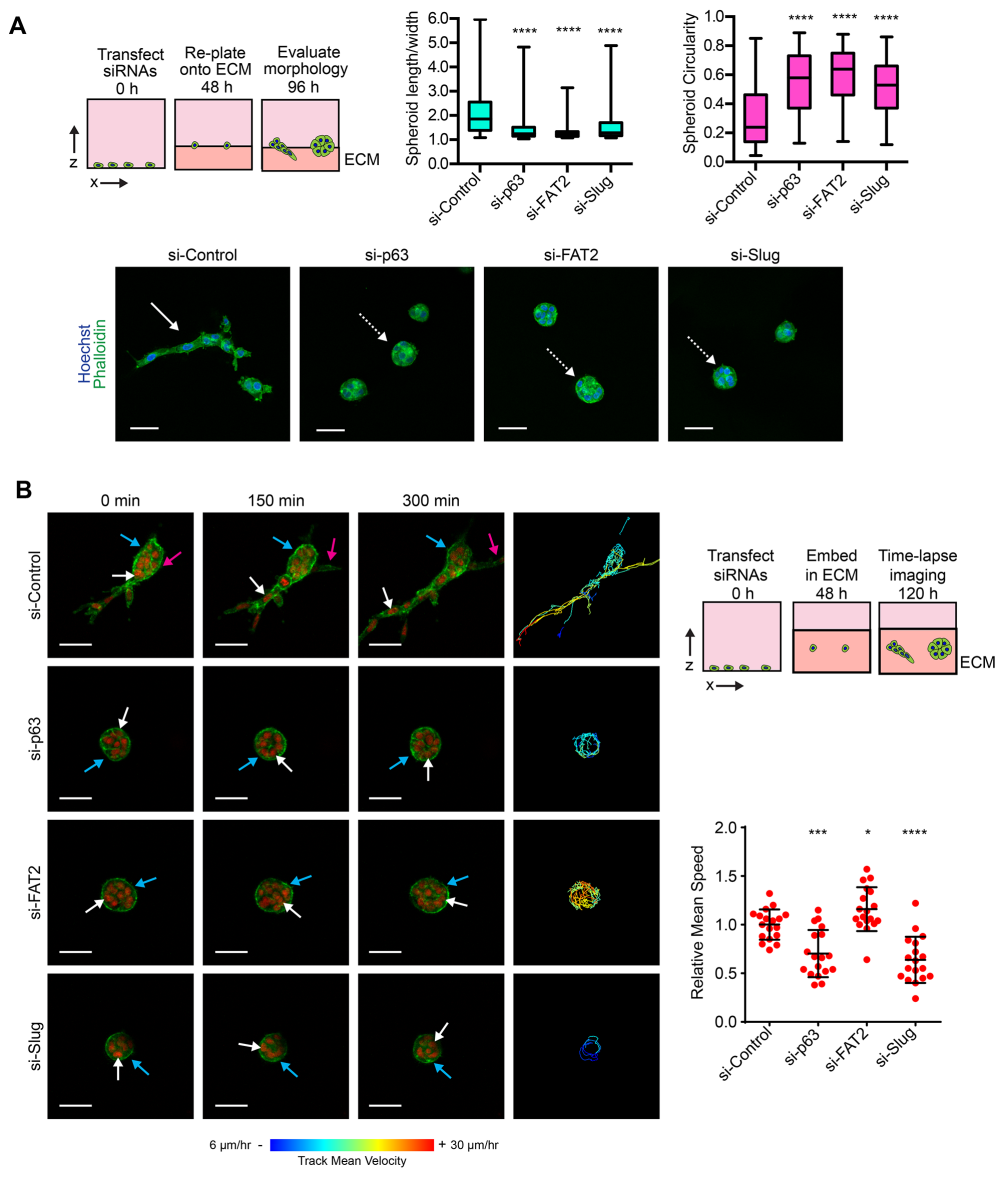
ΔNp63α dependent induction of FAT2 and Slug is necessary for collective invasion **A.** Representative images of MCFDCIS cells transfected with the indicated siRNAs and plated onto a layer of ECM for 48 h. Increased L/W ratio and decreased circularity are characteristic features of invasive spheroids. The solid white arrow indicates an invasive spheroid with the strand-like collective invasion. This invasive spheroid has a L/W ratio >2 and a circularity < 0.25. The dotted white arrows indicate representative non-invasive spheroids. These spheroids have L/W ratios of <1.3 and circularity values of > 0.5. Scale bars = 50 μm. Box and whisker plots show the quantification of spheroid morphology (n >130 spheroids/condition from 4 independent experiments). Whiskers indicate minimum and maximum. ****p< 0.0001, unpaired Student's t-test. Also see Supplementary Figure S5 for additional IF images. **B.** Time-lapse imaging was performed on spheroids formed by MCFDCIS/H2B:mCherry/LifeAct:GFP cells transfected as indicated. Cells were imaged for 6 h total. LifeAct:GFP localizes to regions of F-actin. H2B:mCherry localizes to the nucleus. Solid white arrows indicate the movement of representative cells. Tracking of all cell movement in the spheroids is shown on the right. Each track indicates the movement of an individual cell with blue indicating slower velocity and red indicate faster velocity. Magenta arrows indicate the induction of collective invasion by a new strand of cells away from the spheroid. Blue arrows indicate an area of LifeAct:GFP signal intensity change. Scale bars = 50 μm. Scatter plots show the quantification of the mean cell speed in the spheroids (n= at least 20 spheroids imaged from 3 independent experiments). Error bars indicate SD. *p< 0.05, ***p< 0.001, ****p< 0.0001, unpaired Student's t-test. Also see Supplementary Video S1 for the complete time-lapse imaging and Supplementary Figure S5 for the time-lapse imaging of MCFDCIS spheroids treated with a MEK1/2 inhibitor.

We next focused on understanding the contribution of the ΔNp63α pathway during collective invasion. We previously found that the expression of ΔNp63α and Slug is increased when MCFDCIS cells are induced to collectively invade by mammary fibroblasts in xenograft tumors [17]. ΔNp63α is also increased in cells that invade in explants derived from mouse polyoma virus middle T antigen (PyMT) mammary tumors [18]. Precisely how ΔNp63α promotes collective invasion is not known. The invasion of ΔNp63α expressing breast cancer cells is dependent on extrinsic factors, either reorganization of the ECM by fibroblasts [17] or an increased abundance of Collagen I [18]. Therefore, we investigated how ΔNp63α influenced the collective invasion of MCFDCIS cells into a Collagen I enriched ECM. Control MCFDCIS cells frequently formed multicellular lesions that contained strands of 2 or more cells in a tip-to-tail arrangement (Figure 6A and Supplementary Figure S5A), which is indicative of sprouting collective invasion in organotypic culture [21, 22, 49] and is a characteristic feature of invading neoplastic cells in patient tumors [50–52]. This strand like arrangement of collectively invading cells increases the length-to-width ratio of MCFDCIS spheroids and reduces their circularity (Figure 6A). Conversely, ΔNp63α, Slug and FAT2 depletion reduced the frequency of MCFDCIS collective invasion, with most multicellular lesions forming noninvasive spheroids (Figure 6A and Supplementary Figure S5A). This reduced invasion was further indicated by lower length/width ratio and increased circularity of ΔNp63α, Slug and FAT2 depleted spheroids compared to control spheroids (Figure 6A).

We next performed time-lapse imaging on MCFDCIS cells stably expressing LifeAct:GFP (to visualize F-actin) and H2B:mCherry (to visualize nuclei). In control MCFDCIS spheroids, F-actin-containing protrusions were detected in cells leading the collective invasion of cell strands (Figure 6B and Supplementary Video S1). Additional cells then opportunistically invaded into the ECM following along the paths created by the leading cells in the control spheroids (Figure 6B and Supplementary Video S1). We also detected noninvasive “intraspheroid” motility in control MCFDCIS spheroids (Figure 6B and Supplementary Video S1), which is the migration of cells within the space in the ECM created by proliferative expansion [21]. Interestingly, we also detected intraspheroid movement in the noninvasive spheroids formed by MCFDCIS cells depleted of ΔNp63α, FAT2 and Slug. Notably, the intraspheroid motility speed in FAT2 depleted spheroids exceeded the rate of cell movement in control MCFDCIS spheroids (Figure 6B and Supplementary Video S1). ΔNp63α and Slug depletion modestly reduced the rate of intraspheroid movement (Figure 6B and Supplementary Video S1). However, the ΔNp63α and Slug depleted cells were capable of translocating to new positions in the spheroids independent of cell division (Figure 6B and Supplementary Video S1). The reduction in intraspheroid motility speed in ΔNp63α and Slug depleted spheroids was also not as great as the reduction in speed that was observed in MCFDCIS spheroids treated with a MEK1/2 inhibitor (Supplementary Figure S5B). There were fluctuations in LifeAct:GFP distribution around the surface of the ΔNp63α, FAT2 and Slug depleted spheroids, indicating that F-actin was being formed and severed in areas of cell contact with the ECM (Figure 6B and Supplementary Video S1). Moreover, small transient protrusions into the ECM were detected in ΔNp63α, FAT2 and Slug depleted spheroids (Figure 6B and Supplementary Video S1). However, the protrusions did not mature into the stable type of actin-containing projections that preceded the initiation of collective invasion in control MCFDCIS spheroids (Figure 6B and Supplementary Video S1). Thus, the depletion of ΔNp63α, FAT2 and Slug does not completely perturb all forms of cell movement or entirely disrupt the dynamics of the cytoskeleton. Together, our results indicate that the ΔNp63α dependent induction of FAT2 and Slug is specifically required for the formation of cellular protrusions that initiate collective invasion.

### Increased expression of ΔNp63α, FAT2 and Slug correlates with shorter patient survival time

**Figure 7:**
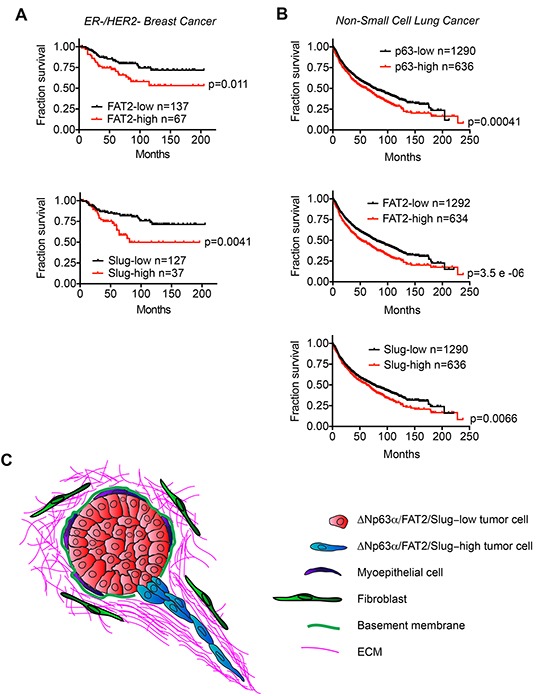
The expression of ΔNp63α, FAT2 and Slug correlates with poor patient outcome **A.** Kaplan-Meier curves show the overall survival of ER-/HER2- breast cancer patients classified based on FAT2 and Slug mRNA expression. Survival differences were compared by log-rank test. **B.** Kaplan-Meier curves show the overall survival of non-small cell lung cancer patients classified based on ΔNp63α, FAT2 and Slug mRNA expression. Survival differences were compared by log-rank test. Analysis of publicly available data sets was performed using KM-Plotter. **C.** Model for how the ΔNp63α dependent induction of FAT2 and Slug may promote collective invasion during tumorigenesis.

The ability of FAT2 and Slug to promote migration suggested that their expression may influence clinically relevant features of tumor progression. We previously found that increased ΔNp63α expression correlated with shorter overall survival time in HER2-/ER- breast cancer patients, which are frequently classified as having BLBC [17]. Therefore, we first evaluated how FAT2 and Slug expression correlated with HER2-/ER- patient outcome. Indeed, consistent with our prior observation evaluating the relationship between elevated ΔNp63α expression and outcome [17], HER2-/ER- patients classified as FAT2-high or Slug-high had lower probability of survival (Figure 7A). Our discovery that the ΔNp63α dependent expression of FAT2 and Slug is necessary for lung SCC migration, and that ΔNp63α is positively correlated with FAT2 and Slug expression in lung cancer patient tumors, suggested that the expression of this ΔNp63α dependent pathway may correlate with non-small cell lung cancer (NSCLC) patient outcome. Indeed, the ΔNp63α-high, FAT2-high and Slug-high NSCLC patient groups all had a shorter overall survival time (Figure 7B). Thus, our results indicate that an elevated expression of ΔNp63α FAT2 and Slug is associated with poor clinical outcome in HER2-/ER- breast cancer and NSCLC patients.

## DISCUSSION

We have found that the transcription factor ΔNp63α can confer cells with migratory traits by inducing the expression of mesenchymal and non-mesenchymal genes with diverse functions. Notably, the ΔNp63α dependent expression of Slug and FAT2 influences the localization of E-cadherin to cell-cell contacts, indicating that ΔNp63α pathway activation blocks the establishment of mature cell-cell adhesion junctions. Moreover, we find that the induction of FAT2 and Slug is specifically required for the formation of cellular protrusions that initiate collective invasion. Importantly, these ΔNp63α dependent pathways may be functional in human tumors and their expression correlates with reduced odds of survival.

### The ΔNp63α dependent induction of a hybrid state is influenced by ΔNp63α binding specificity

Our results provide a more detailed understanding of how ΔNp63α induces a hybrid epithelial/mesenchymal state. Consistent with our previous results [17], our ChIP-seq experiments indicated that ΔNp63α directly interacts with the proximal promoter of Axl and is necessary for Axl expression. We also identified a ΔNp63α binding site in the promoter region of Slug that may regulate Slug expression in BLBC and lung SCC cells. Conversely, at the thresholds used for our analysis, we did not detect ΔNp63α binding associated with other canonical EMT inducing transcription factors, such as Twist [53] or Snail [54]. These results suggest that the nature of the hybrid state induced by ΔNp63α is a function of ΔNp63α binding specificity that allows for the selective and direct regulation of a subset of mesenchymal genes. This mechanism for inducing a hybrid state based on the binding selectivity of a single transcription factor is distinct from previous results demonstrating that hybrid states can be transient unstable intermediates on a committed path towards the conversion to a stable mesenchymal phenotype [55–57]. Moreover, our findings support the concept that there are multiple distinct states induced by components of EMT programs, rather than a simple biphasic switch between a completely epithelial and completely mesenchymal state [22, 56, 58, 59]. In addition, ΔNp63α and Slug are both expressed in mammary stem cells and required for mammary gland re-populating activity in transplantation assays [33, 60]. Whether the selective regulation of Slug and induction of a hybrid state by ΔNp63α is part of a pre-existing biological program that contributes to epithelial stem cell function is an interesting line of future investigation.

### ΔNp63α coordinates the expression of a diverse set of genes that promote cell migration

We have uncovered FAT2, SNCA, CA12, CPNE8 and NEK1 as new targets of ΔNp63α regulation that promote cell migration. Our ChIP-seq and ChIP-qPCR experiments have identified ΔNp63α binding sites in putative regulatory regions associated with these genes, which suggests that ΔNp63α directly promotes their expression. Defining precisely how ΔNp63α binding to these regulatory sites influences pro-migratory expression is an important area of future study. The requirement of FAT2, SNCA, CA12, CPNE8 and NEK1 for ΔNp63α induced motility also suggests new regulatory mechanisms for the control of cell migration. SNCA is a vesicular binding protein [39], CA12 is a carbonic anhydrase [41], CPNE8 is scaffolding protein [38] and NEK1 is a kinase [61, 62]. Based on their known functions, these genes are predicted to act separately in distinct signaling pathways that are integrated with Slug and Axl functions to promote motility, thus revealing the complexity of the ΔNp63α induced signaling network. What little is known regarding FAT2 function suggests it also has a unique contribution compared to these other ΔNp63α regulated genes. FAT2 is a transmembrane protein that was originally detected in the granule cells of the cerebellum [40] and can localize to cell-cell contacts [40, 63]. A single siRNA targeting FAT2 has been reported to suppress the migration of a cutaneous SCC cell line [64]. However, these findings were not supported with a secondary methodology to deplete FAT2 expression in replicate experiments [64]. Thus, whether FAT2 was a bona fide regulator of migration remained unclear. Our combined results showing that (i) distinct FAT2 depletion strategies dramatically reduce MCFDCIS wound closure; (ii) FAT2 is required for migration in multiple cell types; (iii) FAT2 influences E-cadherin recruitment to cell-cell contacts and (iv) FAT2 is specifically required for collective invasion in organotypic culture, demonstrate that FAT2 is a bona fide core regulator of ΔNp63α induced migration. The related FAT family member, FAT1, can promote migration by localizing to the leading free surfaces of migrating cells where it forms a complex that contains ENA and VASP proteins to promote actin polymerization [65, 66]. Given the requirement of FAT2 for collective migration and cell protrusion formation in organotypic culture, it is possible that FAT2 may similarly regulate actin polymerization through protein complex formation.

### Defining the extent of the ΔNp63α regulated pro-migratory signaling network

Additional factors beyond Axl, Slug, FAT2, SNCA, CPNE8, CA12 and NEK1 expression may also be required for ΔNp63α-induced BLBC migration. For instance, we focused on siRNAs that suppressed wound closure to a greater extent than the Axl siRNAs. One or more of the genes targeted by siRNAs that were marginally less potent than the Axl siRNAs at suppressing wound closure may also prove to be part of the ΔNp63α regulated pro-migratory signaling network with further validation. In addition, our functional testing was centered on 37 of 124 genes induced by ΔNp63α in at least 2 BLBC populations. It is possible that one or more of the 87 genes that were not evaluated in this study are direct or indirect targets of ΔNp63α that are essential for migration. The ΔNp63α dependent suppression of gene expression may be necessary for cell motility as well. We established a role for miRNAs in the control of ΔNp63α-dependent cell motility in our previous work demonstrating that miR205 can enhance BLBC migration [17]. We did not identify other known ΔNp63α regulated miRNAs [67] as inducers of migration in our previous wound closure screen, which focused on miRNAs function [17]. However, it is possible ΔNp63α may regulate motility through the induction of miRNAs that were not part of the screen or are necessary for migration in other contexts. ΔNp63α may also suppress miRNAs that act as inhibitors of migration. Previous reports have suggested ΔNp63α promotes migration in other cell types by inducing the expression of genes not found in the set of 124 ΔNp63α target genes we identified [19, 68–70]. Our approach of focusing on genes regulated by ΔNp63α in at least 2 distinct populations, potentially eliminated genes from consideration if their induction was dependent on a different genetic context or they were reliant on exogenous ΔNp63α for expression. Whether the ΔNp63α signaling network we defined cooperates with these genes in other cell populations is important for further investigation.

### ΔNp63α induces invasion in multiple tumor lineages

Our study showed that ΔNp63α regulates migration through inducing Slug and FAT2 expression in a lung SCC population. Notably, ΔNp63α expression also correlated with FAT2 expression across a wide-range of tumors, with Slug also showing a strong correlation in lung SCC, prostate and bladder cancer. These results suggest that ΔNp63α may promote cell migration through the induction of Slug and FAT2 expression, in multiple tumor lineages. Whether the additional ΔNp63α pro-migratory genes we identified are also conserved regulators of ΔNp63α-dependent migration remains to be determined. It is possible that these genes are specifically required for ΔNp63α induced migration in BLBC, and that an alternative process complements ΔNp63α induced gene expression to promote invasion in additional tumor types.

### Regulation of motility by ΔNp63α is dependent on cellular context

Whether ΔNp63α promotes or represses cell migration is likely dependent on cell context and the type of migratory behavior analyzed. Our results have demonstrated that ΔNp63α promotes the migration of hybrid BLBC cells and lung squamous cancer cells. Conversely ΔNp63α can suppress mesenchymal-like cancer migration through the miR205 dependent suppression of ZEB1/2 [27, 28, 71], and the silencing of FAK [72]. Mesenchymal-like cells express low to undetectable levels of ΔNp63α [17], and thus have adopted a ΔNp63α-independent migratory mechanism. Whether other cell types are dependent on ΔNp63α for migration may also be dependent on ΔNp63α abundance, with cells that express low levels of ΔNp63α [73] likely not requiring it for migration.

### ΔNp63α promotes protrusion formation during collective invasion by inducing FAT2 and Slug expression

Our live-imaging experiments provided insight into the nature of the ΔNp63α dependent collective invasion. During the initial induction of invasion, ΔNp63α expressing MCFDCIS cells formed F-actin containing protrusions that extended into the ECM. The nucleus then translocated into the protrusion, resulting in the invasion of the first leading cell. The leading cell then continued invading into the ECM and was followed by additional cells that migrated from the spheroid. This process is similar to the basic features of a leader-follower relationship that is established during various forms of collective invasion that take place during tissue morphogenesis and neoplastic invasion [74]. Cells depleted of ΔNp63α, FAT2 or Slug were capable of moving within the spheroids and formed and severed F-actin, which is a key constituent of invasive protrusions. However, any protrusions that were formed were transient in nature and did not sufficiently extend into the ECM to allow the cells to invade. These results extend on our previous finding that distinct cell signaling pathways are required for collective invasion, but not intraspheroid movement [22] by revealing that ΔNp63α, FAT2 and Slug are specifically required for collective invasion in organotypic culture. During tumor progression in vivo, collective invasion can promote the initial transition from benign noninvasive to malignant invasive growth [17]. Notably, this invasive behavior correlates with an increase in ΔNp63α expression and can be induced by exogenous Slug in orthotopic xenografts [17]. It has recently been shown that completion of an EMT program is not necessary for metastasis in genetically engineered mouse models of breast [75] and pancreatic cancer [76]. The induction of a sustained mesenchymal state can also prevent metastatic outgrowth at distant sites [77, 78]. These findings suggest that metastasis may require the activation of migration programs, such as the hybrid state induced by ΔNp63α, which can promote invasion without triggering the full conversion to a mesenchymal state. Together, our results suggest that the ΔNp63α dependent expression of Slug and FAT2 can induce collective invasion which promotes local dissemination and can potentially lead to metastatic growth (Figure 7C).

### Clinical relevance of ΔNp63α pathway activation

NSCLC and BLBC patients classified as ΔNp63α-, Slug- or FAT2-high had a worse odds of survival compared to the ΔNp63α-, Slug- and FAT2-low patients. In addition, elevated ΔNp63α expression is a defining characteristic of invasive bladder cancer patients with shorter survival times [15, 16]. These combined findings support the further investigation of ΔNp63α, Slug and FAT2 as potential prognostic biomarkers. Our functional studies suggest that an enhanced ability to invade may contribute to the poor outcome observed in the ΔNp63α-high, FAT2-high and Slug-high patient groups. The ability of ΔNp63α to promote invasion may also explain the clinical significance of single nucleotide polymorphism that increases ΔNp63α mRNA levels and is associated breast cancer patient poor outcome [79]. In addition, ΔNp63α and Slug can be required for maintaining a pool of cells with tumor initiating ability [33, 60, 80], which could also contribute to the nature of disease progression and response to treatment. While ΔNp63α, FAT2 and Slug are not likely druggable targets, the further delineation of signaling pathways that regulate ΔNp63α expression, or the determination of which proteins are regulated by Slug and FAT2 to promote invasion, may reveal targetable intervention points that have the potential to increase the survival time of some BLBC, NSCLC and bladder cancer patients.

## MATERIALS AND METHODS

### Cell culture

Cells were cultured at 5% CO_2_, humidified, and at 37°C. MCFDCIS cells (Asterand) were cultured in DME-F12, 5% horse serum, 20 ng/ml EGF, 0.5 μg/ml hydrocortisone, 100 ng/ml cholera toxin, 10 μg/ml insulin and 1X pen/strep. HCC1313 cells [81] were a gift from John Minna (UTSW). All cell lines were validated by Powerplex genotyping before use. HCC1313 cells were cultured in 5% FBS, RPMI. MCFDCIS cells stably expressing LifeAct-GFP (Addgene #46356, gift from Iain Cheeseman) and PGK-H2B:mCherry (Addgene #21217, gift of Mark Mercola; [82]) were generated as described [21]. Growth factor reduced Matrigel (BD Biosciences, 10-12 mg/ml stock concentration) and bovine dermal collagen I (BD Biosciences) were used for organotypic culture experiments. The antibodies used are listed in Supplementary Table S6.

### ChIP-seq

ChIP was performed as described [17] with α-p63α antibody (Santa Cruz, H-129). Briefly, cross-linking was performed with 1% formaldehyde at room temperature for 15 min and lysed in PIPES hypotonic buffer (5 mM PIPEs pH8, 85 mM KCL, 0.5% NP40) for 30 min at 4°C. After pelleting, lysates were resuspended in RIPA buffer (10 mM Tris-HCL pH 7.5, 150 mM NaCL, 1 mM EDTA, 1% sodium deoxycholate, 0.1% SDS, 1% NP-40 and protease inhibitors) and rotated overnight at 4°C. Chromatin was sheared by sonication and pre-cleared with ProteinA sepharose beads that had been pre-blocked with sheared salmon sperm DNA and BSA. Cleared chromatin samples were then incubated overnight with anti-p63α antibody after which ProteinA sepharase beads were added for 2 h and pelleted by centrifugation. Bead-antibody complexes were then washed with Wash Buffer 1 (150 mM NaCl, 20 mM Tris-HCl pH 8.1, 2 mM EDTA, 0.1% SDS and 1% TritonX-100), Wash Buffer 2 (500 mM NaCl, 20 mM Tris-HCl pH 8.1, 2 mM EDTA, 0.02% SDS and 1% TritonX-100), Wash Buffer 3 (250 mM LiCl, 10 mM Tris-HCl pH 8.1, 1 mM EDTA, 1% sodium deoxycholate and 1% NP-40), Wash Buffer 4 (10 mM Tris-HCl pH7.9 and 1 mM EDTA) and resuspended (10 mM Tris-HCl pH7.9, 1 mM EDTA, 0.5% SDS). The DNA was then purified using QiaQuick PCR purification columns (Qiagen) following the manufacturer's protocol.

ChIP was performed twice on different days using unique MCFDCIS lysates. Both experiments were subjected to next-generation sequencing. ChIP-seq libraries were prepared with a KAPA HTP Library Preparation Kit. Sequencing was performed using an Illumina HiSeq 2500 platform using 50SR SBS v3 reagents at the UT Southwestern McDermott Center Next Generation Sequencing Core. Single-end reads of 51 bp were generated. After mapping reads to the human genome (hg19) using Bowtie2 (version 2.2.3, [83]) with parameter “--sensitive”, we performed filtering by removing alignments with a mapping quality <10 and by removing duplicate reads identified by Picard MarkDuplicates (version 1.92, http://broadinstitute.github.io/picard). Enriched regions (peaks) were identified using MACS2 (version 2.0.10.20131216, [84]) with a q-value cut-off of 0.05 (GSE72009). There were 15,239 recurrent ΔNp63α binding peaks identified from 2 independent experiments. The 2 biological replicates were checked for reproducibility by irreproducible discovery rate analysis [85]. Peak regions were annotated by HOMER [86]. Motif analysis was performed using HOMER with a region size of 50 bp from peak centers and motif lengths of 8, 10 or 12. ngs.plot [87] was used to plot the ratio of the ΔNp63α signal compared to the input signal across all genes in the human genome.

### Identification of peaks in enhancer regions

Enhancer regions containing H3K4me1 and H3K4me2 modifications were defined in human mammary epithelial cells (HMECs) by ENCODE [85] and used to further annotate peaks regions by BEDOPS (version 2.4.2) [88]. Peaks overlapping with at least one base pair with the enhancer regions defined in HMECs were classified as potential enhancers. The relative enrichment of peaks in enhancer regions of HMECs, epidermal keratinocytes and lung fibroblasts defined by ENCODE was calculated using ngs.plot (version 2.47) [87].

### Identification of ΔNp63α activated genes

The mRNA expression was determined using Human HT-12 v4 Expression BeadChips (Illumina Inc.). Data was processed with a model-based background correction approach [89], quantile-quantile normalization and log2 transformation. The raw data was reported in [17] and is available at the GEO (GSE58643, GSE62569). To identify genes that are regulated by ΔNp63α expression, we determined which genes were ≥ 2-fold reduced by ΔNp63α siRNA transfection with a p ≤0.05 in MCFDCIS and HCC1806 cells. As an additional parameter, we only considered genes with probe read values of ≥40 in the control MCFDCIS and HCC1806 cells. This list was manually curated to remove genes that had a non-concordant reduction in signal across multiple probes in response ΔNp63α siRNA transfection. We then determined which of the 124 ΔNp63α-dependent genes identified using these thresholds had ΔNp63α peaks (scores >100) that were located within 2 kb of their TSS or associated enhancer regions as described above.

### Transfection of siRNAs

Cells were transfected with 50 nM of siRNA using RNAiMax transfection reagent (Invitrogen) for 24-48 h. The siRNAs were from Dharmacon (designated siRNA #1) and Sigma (designated siRNA #2). Cells in all conditions designated as “Control” were transfected with a pool of siRNAs that does not target human genes. Pools of at least 3 siRNAs were used to dilute potential off-target effects. The details of the sequences the siRNAs used are in Supplementary Table S7.

### Wounding assay

Experiments were performed as described [17]. Wounds were generated with 96-pin wounding tool (AFIX96FP6, V&P Scientific). MCDCIS cells were wounded with 1.68 mm diameter pins (FP6-WP) and a monolayer wounding library copier that introduces a wound length of 4.5 mm (VP 381NW 4.5, V&P Scientific). HCC1313 cells were wounded 1.014 mm diameter pins (FP4-WP) and a monolayer wounding library copier that introduces a wound length of 4.1 mm (VP 381NW 4.5, V&P Scientific). Immediately after wounding wells were washed twice with media to remove debris. After wounding, MCFDCIS cells were cultured in MCFDCIS media (described above). HCCC1313 cells were cultured in 1% FBS, 5 ng/ml EGF in RPMI. Twenty-four hours after wounding, cells were fixed in 2% formalin and stained with phalloidin-546 and Hoechst. Wounds were imaged on a BD Pathway 855 microscope with a 10x objective (Olympus, UPlanSApo 10x/0.40, ∞/0.17/FN26.5). Images were acquired as 6x4 montages. A custom designed analysis protocol was generated using Pipeline Pilot software, which used a threshold of pixel signal intensity to determine the amount of empty space in each well not occupied by cells [17]. The amount of empty space in the well was inversely proportional to the extent of wound closure. For the parallel testing of 37 siRNAs in triplicate, wounding activities were normalized to internal controls and z-scores were calculated (z score = siRNA Activity Score – Mean Activity score of mock transfected cells)/ (Standard deviation of mock transfected cells).

### ChIP qPCR

ChIP was performed as described for the ChIP-seq experiments using anti-p63α antibody (Santa Cruz, H-129) and normal rabbit IgG (Santa Cruz, 2027) antibodies. Primers are listed in Supplementary Table S8. PCR was performed using BioRAD iTaq Universal SYBR Green Master Mix. Temperatures and times for RT-PCR followed manufacturer's protocol for Applied Biosystems 7500 Real-Time PCR System. The percent input was determined as (2^(Average Ct for Input/ Ct of sample)) × (% Input used in qPCR) × 100.

### Quantitative real-time PCR

A list of primers used is included in Supplementary Table S8. Total RNA was isolated using RNAeasy purification columns (Qiagen) and converted to cDNA using the iScript cDNA Synthesis Kit (Bio-Rad). Applied Biosysems TaqMan Gene Expression Assays were performed with 20 ng of cDNA was amplified with Applied Biosystems 2X TaqMan using an Applied Biosystems 7500 Real-Time PCR System. GAPDH and specific transcript levels for each transfection condition were measured in triplicate. The ΔΔCT method was applied to quantify relative gene expression [90].

### Immunoblotting

Cells were lysed in RIPA buffer supplemented with a protease inhibitor cocktail (Calbiochem) as described [17]. Equal amounts of protein were separated by SDS-PAGE, transferred to Immobilon-FL polyvinylidene fluoride (PVDF) transfer membrane (Millipore), and immunostained. Immunoblots were visualized using an Odyssey infrared scanner (LI-COR).

### Immunofluorescence

MCFDCIS cells were reverse transfected with 50 nM of siRNA in 8 well chamberslides (BD Biosciences) coated with poly-l-lysine at a density of 2500 cells per well. After 48 h, cells were fixed in formalin and immunostained as described [91]. Images were acquired on a Zeiss LSM700 confocal microscope in TIFF format. Quantification of E-cadherin signal intensity was performed using the ‘Measure…’ function of Image J to determine the pixel intensity in a region of interest at the cell-cell boundaries. The results were then normalized to the mean E-cadherin signal intensity for all cell-cell boundaries analyzed in the control cells for each experiment. Images were arranged using Illustrator 6 (Adobe).

### Organotypic culture experiments

Cells were transfected with siRNAs for 48 h and then either re-plated onto ECM or embedded in ECM consisting of 5 mg/ml Matrigel and 2.1 mg/ml Collagen I. Cultures were overlaid with MCFDCIS growth media containing 2% Matrigel. For analysis of spheroid morphology, 48 h after plating onto ECM, the dimensions of at least 40 spheroids per condition for experiment were evaluated. Images of phalloidin-stained cultures were thresholded for brightness to trace cell edges, and then the ‘Analyze…’ function of ImageJ was used to obtain length and width and circularity measurements. Time-lapse imaging of spheroid invasion was performed 72 h after transfected cells were embedded in ECM using a Zeiss LSM700 laser scanning confocal microscope enclosed in a 37°C chamber supplemented with humidified CO_2_ (Solent). Images were acquired with a 10X or 20x objective (Zeiss) using ZenBlack software (Zeiss) and analyzed with Image J.

### Gene expression analysis in patient tumors

The results shown here are based upon data generated by the TCGA Research Network: http://cancergenome.nih.gov/”. The cBioPortal web resource (http://www.cbioportal.org) [92, 93] was used to analyze correlations in gene expression in patient tumors. The provisional TCGA datasets corresponding to invasive breast cancer [42], lung squamous cell carcinoma [43], lung adenocarcinoma [44] bladder cancer [45], prostate cancer [46] and pancreatic ductal adenocarcinoma were used for determining correlations in gene expression. RNA-seq data was used for breast cancer, lung adenocarcinoma and bladder cancer patients. Microarray data was used for lung squamous cell cancer patients.

### Patient survival analysis

Analysis of breast cancer and NSCLC patient survival times was performed using the KM-plotter meta-analysis database [94]. Patients were stratified into “high” and “low” groups based on the upper tertile of gene expression. Estrogen receptor (ER) and progesterone receptor (PR) status was judged by mRNA expression. Survival differences were compared by log-rank test. p63 probe= 209863; FAT2 probe= 208153 and Slug probe= 213139 were used.

### Statistical methods

Data was analyzed by two-tailed Student's t-test (Graphpad Prism) with the exception of patient survival differences, which were analyzed by log-rank test. P-values < 0.05 were considered significant.

## SUPPLEMENTARY FIGURES, TABLES AND VIDEO




















