# Supplementary material for: ΔNp63α induces the expression of FAT2 and Slug to promote tumor invasion
Source: Oncotarget. 2016 Apr 12;7(19):28592–611. doi: 10.18632/oncotarget.8696 (PMC5053748; doi:10.18632/oncotarget.8696)
Supplement: Supplementary file 1 [file oncotarget-07-28592-s001.pdf]

SUPPLEMENTARY FIGURES, TABLES AND VIDEO

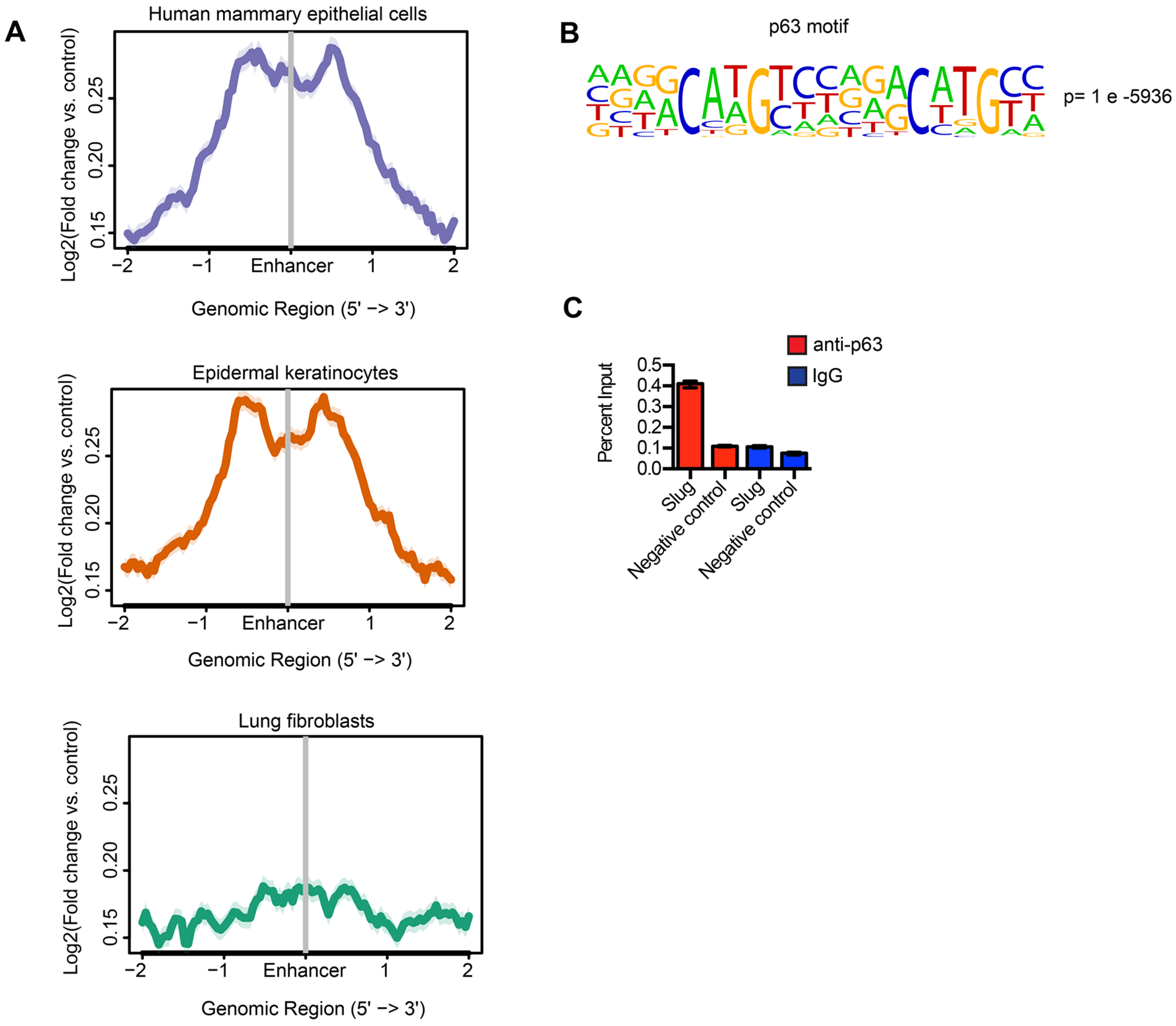

Supplementary Figure S1: A. ΔNp63α binding relative to input sample ±2 kb from the center of enhancer regions defined by ENCODE. ΔNp63α binding was enriched in enhancer regions defined in ΔNp63α expressing human mammary epithelial cells (HMECs) and ΔNp63α expressing keratinocytes, as shown in the top and middle plots. ΔNp63α binding was not enriched in the enhancer regions defined in ΔNp63α deficient pulmonary fibroblasts, as shown in the bottom plot. **B.** Analysis of known binding motifs shows the established "CNNG" ΔNp63α binding motif is the top enriched motif in ΔNp63α bound sequences. **C.** ChIP-qPCR performed on the ΔNp63α binding site associated with Slug indicated with a green arrow in Figure 1F. Red bars indicate samples immunoprecipitated with α-p63α antibody. Blue bars indicate samples immunoprecipitated with rabbit IgG. The negative control is a genomic region that does not contain a ΔNp63α binding site. Graph shows mean ± range of a representative experiment analyzed in triplicate.

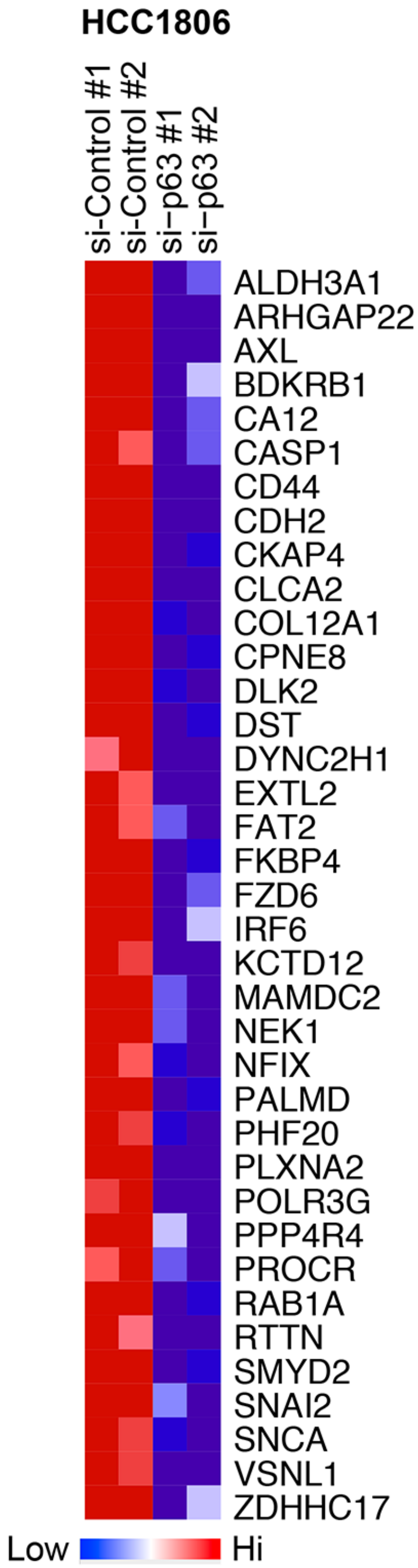

Supplementary Figure S2: Heatmap shows the expression of 37 genes with associated ΔNp63α binding sites in HCC1806 cells transfected with a ΔNp63α siRNA pool. Biological replicates are shown.

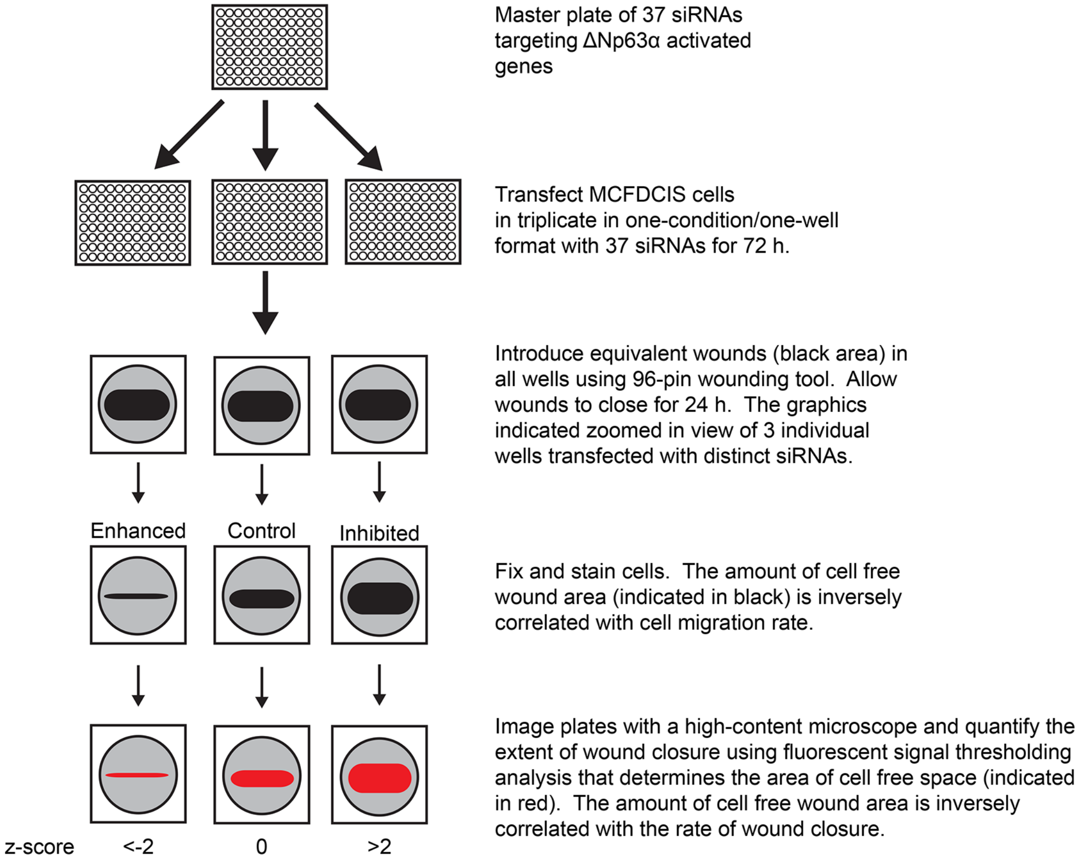

Supplementary Figure S3: The model figure shows the procedure for determining how sRNAs influenced MCFDCIS wound closure.

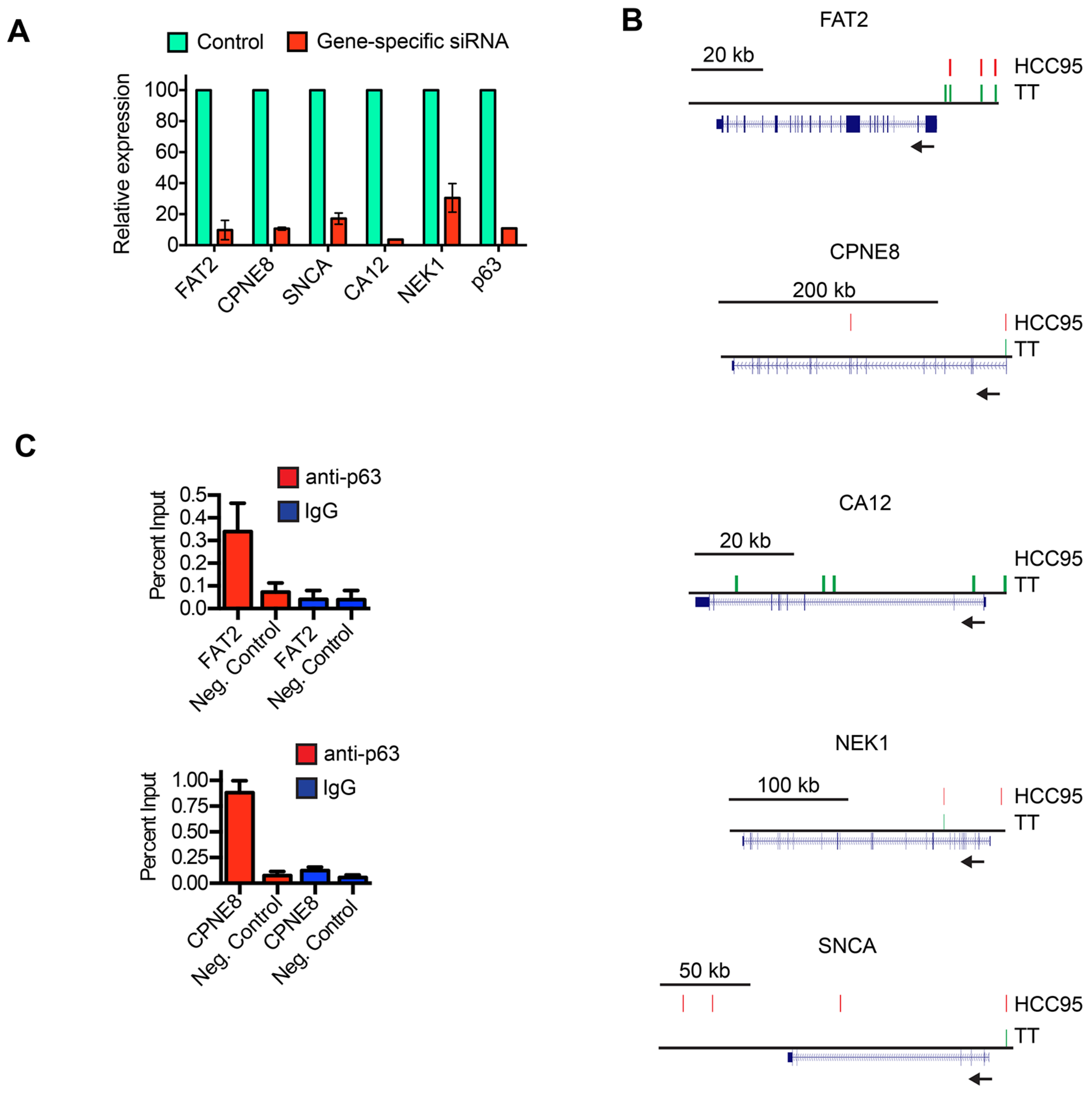

Supplementary Figure S4: A. Confirmation that the siRNA pool #2 from Figure 2C corresponding to the indicated genes depletes expression, as determined by qPCR. Graph shows mean ± range from 2 independent experiments. **B.** ΔNp63α binding sites in squamous carcinoma cells were determined using peak calls from GSE46837 and are indicated by red bars (HCC95) and green bars (TT). **C.** ChIP qPCR performed on ΔNp63α binding sites associated with FAT2 and CPNE8 indicated with "x" in Figure 2G. Red bars indicate samples immunoprecipitated with α-p63α antibody. Blue bars indicate samples immunoprecipitated with rabbit IgG. The negative control (neg. control) is a genomic region that does not contain a ΔNp63α binding site. Graph shows mean + range of 2 independent experiments.

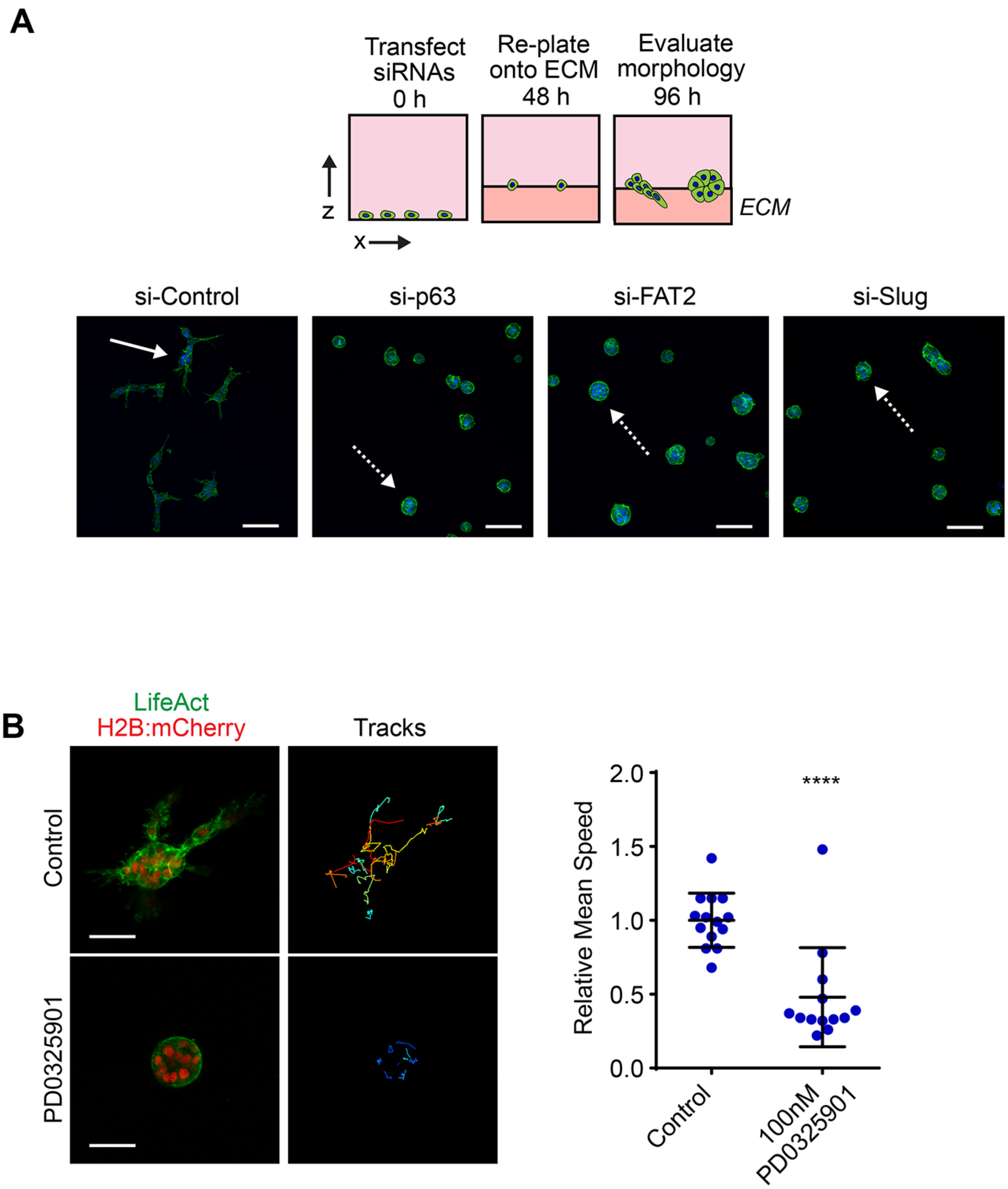

Supplementary Figure S5: A. Representative images of MCFDCIS cells transfected with the indicated siRNAs and grown on ECM for 48 h. The solid white arrow indicates an invasive spheroid as indicated with the strand-like invasion. The dotted white arrows indicate representative non-invasive spheroids. Scale bars = 50 µm. **B.** Representative images from time-lapse imaging performed on MCFDCIS/H2B:mCherry/LifeAct:GFP spheroids treated with diluent or 100 nM PD0325901 (MEK1/2 inhibitor). Cells were imaged for 6 h total. Tracking of all cell movement in the spheroids is shown on the right. Each track indicates the movement of an individual cell with blue indicating slower velocity and red indicate faster velocity. Scale bars = 50 µm. Scatter plots show the quantification of the mean cell speed in the spheroids (n= at least 20 spheroids imaged from 3 independent experiments). Error bars indicate SD. ****$p < 0.0001$, unpaired Student's t-test.

Supplementary Table S1: Genes induced by ΔNp63α in MCFDCIS and HCC1806 cells. Genes decreased ≥ 2-fold with $p < 0.05$ in ΔNp63α depleted cells.

See Supplementary File 1

Supplementary Table S2: Genes suppressed by ΔNp63α in MCFDCIS and HCC1806 cells. Genes increased ≥ 2-fold with $p < 0.05$ in ΔNp63α depleted cells.

See Supplementary File 2

Supplementary Table S3: Genes induced by ΔNp63α in MCFDCIS and HCC1806 cells that have ΔNp63α binding sites located within 2 kb of TSSs or enhancer regions. The list of genes from Supplementary Table S1 that have associated ΔNp63α binding sites.

See Supplementary File 3

Supplementary Table S4: The location of the ΔNp63α binding sites associated within ΔNp63α activated genes. Genomic location of the ΔNp63α binding sites associated with the 37 genes targeted by siRNAs tested for a wound closure phenotype in Figure 2B. The ratio of gene expression in control siRNA and ΔNp63α siRNA transfected MCFDCIS and HCC1806 cells is also included.

See Supplementary File 4

Supplementary Table S5: The z-scores from the wounding closure assay performed with siRNAs targeting the 37 ΔNp63α activated genes with associated ΔNp63α binding sites.

See Supplementary File 5

Supplementary Table S6: List of antibodies used.

See Supplementary File 6

Supplementary Table S7: List of siRNA sequences used.

See Supplementary File 7

Supplementary Table S8: List of primers used for qPCR and ChIP-qPCR.

See Supplementary File 8

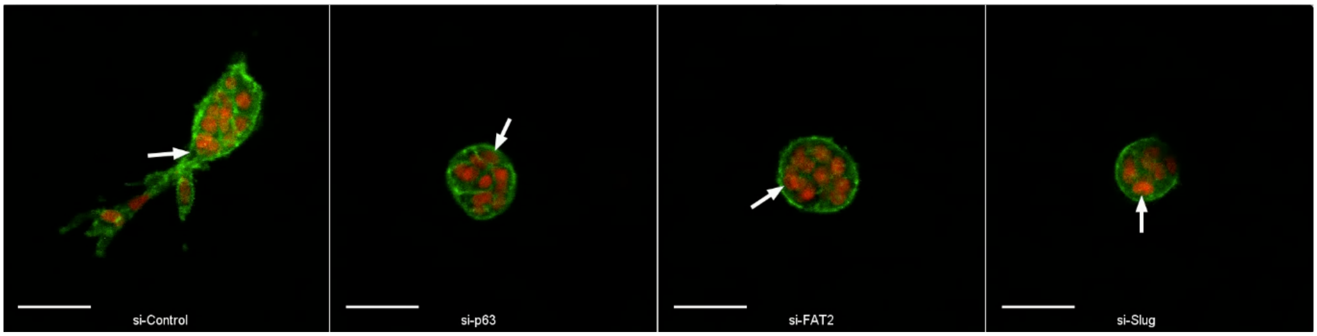

Supplementary Video S1: Time-lapse imaging of spheroids formed by MCFDCIS/H2B:mCherry/LifeAct:GFP cells transfected with control, ΔNp63α, FAT2 or Slug siRNAs. The video corresponds to the images shown in Figure 6B. Images were acquired at 15 min intervals for 7 h.
